# Enhanced RGB-D Mapping Method for Detailed 3D Indoor and Outdoor Modeling

**DOI:** 10.3390/s16101589

**Published:** 2016-09-27

**Authors:** Shengjun Tang, Qing Zhu, Wu Chen, Walid Darwish, Bo Wu, Han Hu, Min Chen

**Affiliations:** 1 State Key Laboratory of Information Engineering in Surveying Mapping and Remote Sensing, Wuhan University, 129 Luoyu Road, Wuhan 430079, China; Shengjun.tang@whu.edu.cn; 2 State-Province Joint Engineering Laboratory of Spatial Information Technology for High Speed Railway Safety, Chengdu 610031, China; 3 Faculty of Geosciences and Environmental Engineering, Southwest Jiaotong University, Chengdu 610031, China; huhan@whu.edu.cn (H.H.); minchen@home.swjtu.edu.cn (M.C.); 4 Collaborative Innovation Center for Geospatial Techneology, 129 Luoyu Road, Wuhan 430079, China; 5 Department of Land Surveying & Geo-Informatics, The Hong Kong Polytechnic University, Hung Hom 999077, Hong Kong, China; wu.chen@polyu.edu.hk (W.C.); w.darwish@connect.polyu.hk (W.D.); bo.wu@polyu.edu.hk (B.W.)

**Keywords:** indoor modeling, RGB-D camera, depth, image, camera pose, registration

## Abstract

RGB-D sensors (sensors with RGB camera and Depth camera) are novel sensing systems that capture RGB images along with pixel-wise depth information. Although they are widely used in various applications, RGB-D sensors have significant drawbacks including limited measurement ranges (e.g., within 3 m) and errors in depth measurement increase with distance from the sensor with respect to 3D dense mapping. In this paper, we present a novel approach to geometrically integrate the depth scene and RGB scene to enlarge the measurement distance of RGB-D sensors and enrich the details of model generated from depth images. First, precise calibration for RGB-D Sensors is introduced. In addition to the calibration of internal and external parameters for both, IR camera and RGB camera, the relative pose between RGB camera and IR camera is also calibrated. Second, to ensure poses accuracy of RGB images, a refined false features matches rejection method is introduced by combining the depth information and initial camera poses between frames of the RGB-D sensor. Then, a global optimization model is used to improve the accuracy of the camera pose, decreasing the inconsistencies between the depth frames in advance. In order to eliminate the geometric inconsistencies between RGB scene and depth scene, the scale ambiguity problem encountered during the pose estimation with RGB image sequences can be resolved by integrating the depth and visual information and a robust rigid-transformation recovery method is developed to register RGB scene to depth scene. The benefit of the proposed joint optimization method is firstly evaluated with the publicly available benchmark datasets collected with Kinect. Then, the proposed method is examined by tests with two sets of datasets collected in both outside and inside environments. The experimental results demonstrate the feasibility and robustness of the proposed method.

## 1. Introduction

Detailed 3D modeling of indoor and outdoor environments is an important technology for many tasks such as indoor mapping, indoor positioning and navigation, and semantic mapping [1]. Traditionally, there are two main approaches to close-range 3D modeling—terrestrial laser scanning (TLS) and close-range photogrammetry. With TLS technology, the obtained 3D point clouds contain detailed structural information and are well suited for frame-to-frame alignment. However, TLS lacks valuable visual information that is contained in RGB images. Although RGB images are easily captured with off-the-shelf digital cameras and their rich visual information can be used for loop closure detection [2,3], it is hard to obtain enough points for dense modeling through regular photogrammetric techniques, especially in dark environments and poorly textured areas [1,4,5,6].

Recently, the advent of RGB-D sensors (such as the Kinect or the Structure sensor) has led to great progress in dense mapping and simultaneous localization and mapping (SLAM) [7,8,9,10]. The remarkable advantages of these systems lie in the high mobility and low cost. However, RGB-D sensors have some significant drawbacks with respect to dense 3D mapping. These sensors only allow measurement ranges of a limited distance and a limited field of view. This may cause tracking loss due to lack of the spatial structure needed to constrain ICP (iterative closest point) alignments [1]. Particularly, as the random error of the measurement depth increases with distance from the sensor, only the data acquired within the range from 0 to 3 m to the sensor can be used for mapping applications [11]. The RGB-D sensors capture RGB images along with per-pixel depth images, which enables the estimation of the camera poses and the scene geometry with image-based algorithms such as SLAM or structure-from-motion (SFM). The 3D scene recovered from RGB image sequences is expected to have a larger and longer range than that from the depth sensors, but the motion between frames can only be recovered up to a scale factor, and the errors in tracking motion can accumulate over time during frame-to-frame estimation [5,12]. The RGB image-based and depth-based methods for 3D modeling each has its own advantages and disadvantages, but a more fundamental solution is desired for enhancing the capability of RGB-D sensors to perform indoor mapping [13].

Traditionally, only 3D information from depth sensor is used for scene modeling, we introduce a novel approach for geometric integration of depth scene and RGB scene to enhance the mapping system of RGB-D sensors for detailed 3D modeling of large indoor and outdoor environments. The 3D scene produced from the RGB images can be innovatively used as supplement to the 3D scene produced by the depth sensors. The method could not only enlarge the measurement range of RGB-D sensors, but also enhance scene details where is lack of depth information.

This paper is organized as follows. First, by presenting a literature review on the modeling approaches with RGB-D sensors developed to date, we give a general description of the device components and the working mechanism of the RGB-D system. The procedure involved in our enhanced RGB-D mapping approach is also briefly introduced. Second, a precise calibration methodology, for both the RGB camera and the infrared (IR) camera, is then presented in detail. Third, a global optimization model is used to improve the accuracy of the camera pose, decreasing the inconsistencies between the depth frames, and we elaborate the refined relative motion estimation method for RGB images sequence and the robust geometric registration methodology for depth scene and RGB scene is then presented. The results of experimental analyses involving datasets collected both outside and inside are used for experimental analysis. Finally, concluding remarks are presented and discussed.

## 2. Literature Review

Recently, the advent of RGB-D sensors (such as the Kinect or the Structure sensor) has led to great progress in dense mapping and in simultaneous localization and mapping (SLAM). However, efficient means have to be developed to utilize the high frame-rate and high resolution images provided by such sensing modalities. As an incremental approach has been typically used for scene modeling in RGB-D sensor system, in which each local frame of data is aligned to a cumulative global model, so such an approach may result in an inconsistent model [14]. Therefore, most research focuses on improving registration accuracy between frames.

Newcombe et al. (2011) proposed the KinectFusion method, which incrementally registers RGB-D frames. As this method accumulates drift during the mapping procedure, the KinectFusion can be applied only in small workspace mapping [15]. Du et al. (2011) introduced a mobile system that runs in real-time on a laptop. Color and depth are jointly used to achieve robust 3D registration. However, some manual interactions should be involved [16]. Henry et al. (2012) proposed an improved registration method denoted as RGB-ICP to incorporate visual information into the ICP algorithm for image registration [17], and this method can improve the alignment accuracy to a certain extent. However, the final models were still broken, and lacked of details in some regions. The authors suggested that it would be helpful to apply a visualization technique such as PMVS (patch-based multi-view stereo) to enrich the indoor model. Engelhard et al. (2011) [18] presented an approach, which is similar to the work of Henry et al. (2012) [17]. Instead of SIFT, this approach applies SURF for feature detection [18]. Khoshelham et al. (2013) presented a Frame alignment method by assigning weights of 3D points correspondences based on the theoretical random error of individual points. However, the method completely relied on the visual features, emphasizing the importance of a fine registration step extracted from the depth images to generate accurate point clouds from RGB-D data [19]. Based on the method of weighting the 3-D points, Santos et al. (2016) introduced a refined mapping method, robust coarse-to-fine registration method. The loop-closure detection and a global adjustment of the frames sequences are used to improve the consistency of the frames sequences [20]. Endres et al. (2014) applied a similar approach, using the RANSAC (RANdom Sample Consensus) method to estimate the transformations between associated key points, and then generated a volumetric 3D map of the environment [21]. In this approach, Endres et al. concentrated mainly on SLAM rather than scene modeling. Stuckler and Behnke (2012) presented an approach for scene modeling and pose tracking that used RGB-D cameras [22]. They conducted two experiments in the small range to evaluate the performance of the registration. Their experiments showed that although the improvement of depth alignment could enlarge the modeling range of the sensor significantly, the absolute distance limitation may still cause trouble when modeling a large-scale indoor scene with a high, arched roof, like the airport terminal or church. Similar to these methods, a kind of multi feature points matching algorithm is proposed for loop closing detection in RGB-D SLAM by combining appearance and local geometric constraints [23]. Chow et al. (2014) [24] introduced a mapping system that integrated a 3D terrestrial LiDAR system with a MEMS IMU and two Microsoft Kinect sensors to map indoor urban environments. A point-to-plane ICP was used to minimize the reprojection error of the infrared camera and projector pair in an implicit iterative extended Kalman filter (IEKF). However, this system is not handheld and its cost would be much higher than single RGB-D sensors.

In the previous developments, only feature points extracted from RGB image were used as a constraint to improve the pose accuracy of depth frames. Although it can merge the entire depth frame well, the final model is completely generated from the depth frame. As presented by Khoshelham and Elberink (2012), only the data obtained within the distance of 1 to 3 m from the depth sensor can be used for mapping [11]. Therefore, the problem associated with measured range limitation still cannot be solved when modeling a scene with a high, arched roof. In addition, the depth sensors capture depth information based on the concept of structured light pattern and time-of-flight, and the measurement is highly related to the material and structure of objects. It would cause “details lost” when modeling objects with smooth surfaces or low reflection certain materials or scene structures which do not reflect infra-red (IR) light, very thin structures or surfaces at glancing incidence angles. Meanwhile, the device will also experience motion blur (like any camera) under fast moving condition, which can also lead to missing data. However, in computer vision, many approaches to Structure from Motion (SfM) are used for 3D scene reconstruction. They allow the production of high quality 3D models by using unordered image collections that depict a scene or an object from different viewpoints [25]. RGB image-based modeling could create 3D models from a collection of images based on visual features instead of material and structure of objects [26,27,28]. The corresponding RGB image sequences generated from RGB-D sensors may not only be used for depth frame registration but also be used to provide extra 3D information for the unmeasured areas including far range scenes and regions with holes.

In this paper, we intended to innovatively integrate the 3D scene generated from image-based modeling method and the 3D scene from depth images for scene modeling. 3D scene from RGB images can not only enlarge the measurement distance of the RGB-D sensors but can also serve as a good supplement to 3D scene from depth images.

Compared with previous works, this paper presents three key novelties. First, a precise calibration for both of IR and RGB cameras is demonstrated. The full set of calibration data for external and internal parameters as well as the relative pose between RGB camera and IR camera can be obtained. Second, a novel false matches rejection method is presented by combining the depth information and the initial pose parameters from the RGB-D sensor. Third, the image-based modeling method is innovatively incorporated to enhance the mapping system of RGB-D scenes. A global optimization model is used to improve the accuracy of the camera pose, decreasing the inconsistencies between the depth frames. In order to eliminate the geometric inconsistencies between 3D scene from RGB images and depth scene, the scale ambiguity problem encountered during the pose estimation with RGB image sequences can be resolved by integrating the depth and visual information. A robust rigid-transformation recovery method is developed to register 3D scene from RGB images to depth scenes.

## 3. Enhanced RGB-D Mapping for Indoor Environments

### 3.1. Overview of the Enhanced RGB-D Mapping System

**Figure 1:**
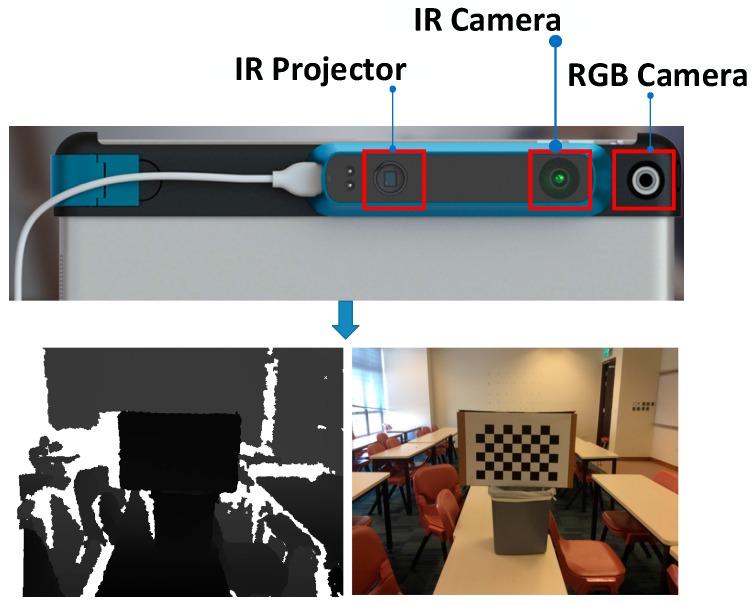
(**top**) The hardware scheme of the RGB-D sensor (sensor with RGB camera and Depth camera); (**bottom left**) the acquired depth image; and (**bottom right**) the acquired RGB image.

The RGB-D sensor system used in this research contains two sensors: one RGB camera, and one IR sensor called “Structure sensor”. The IR sensor is combined with an IR camera and an IR projector. This sensor system is highly mobile, and can be attached to an iPad, iPhone, or other mobile instrument. The system can capture 640 × 480 registered RGB images and depth images at 30 frames per second. Figure 1 shows its hardware structure. The lower panels of Figure 1 show a sample frame observed with the RGB-D sensor. The white part of the depth image indicates that no depth information is measured due to certain materials or scene structures that do not reflect infrared (IR) light, very thin structures or surfaces at glancing incidence angles.

**Figure 2:**
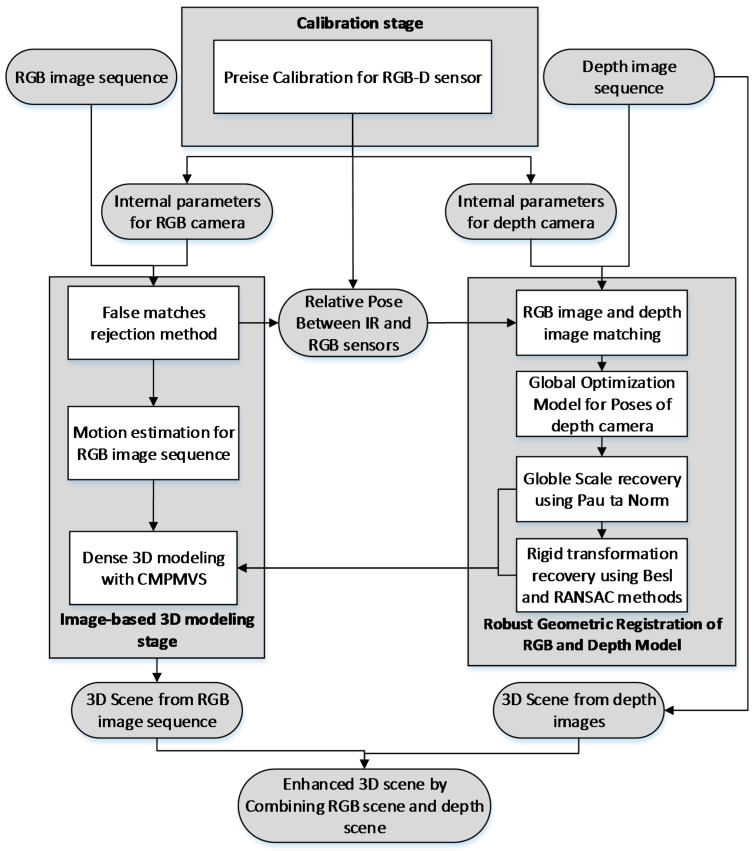
Flowchart of the enhanced RGB-D mapping approach.

The proposed enhanced RGB-D mapping system can be divided into three stages: the calibration stage, the image-based 3D modeling stage and the robust geometric registration of RGB and depth model stage, as illustrated in Figure 2. First, a precise calibration for both the RGB camera and the IR camera is conducted, and the results of calibration stage is the full set of calibration data for external and internal parameters as well as the relative pose between RGB camera and IR camera. These parameters could be used in the robust registration process. Second, a refined image-based modeling method is used for 3D scene reconstruction from RGB images. A novel false matches rejection method is used to minimize the false matches during feature matching process. A key frames selection method is used to ensure sufficient overlapping between the candidates and the previous key frame. Third, in the stage of robust geometric registration of RGB and depth model, a global optimization model is used to improve the accuracy of the camera pose, decreasing the inconsistencies between the depth frames. The accurate global scale factor is recovered for RGB sequences combining RANSAC and Pau Ta Norm and the rigid geometric transformation between RGB model and depth model is robust calculated using Besl and RANSAC method. Finally, according to the registration parameters, the 3D scene from RGB images can be registered to the 3D scene from depth images well.

### 3.2. Precise Calibration for RGB-D Sensors

The main concept of camera calibration is based on the pinhole camera model shown in Equation (1), which illustrates the relationship between the image point and the corresponding ground point as a function of the camera’s internal and external parameters. Both IR and RGB cameras can use this model.

(1) $$s\left[\begin{array}{c} u\\ v\\ 1 \end{array}\right]=K\left[R|t\right]\left[\begin{array}{c} X\\ Y\\ \begin{array}{c} Z\\ 1 \end{array} \end{array}\right]$$

where s is the scale factor, u, v are the image points coordinates in pixels, $K=\left[\begin{array}{ccc} f_{x} & 0 & c_{x}\\ 0 & f_{y} & c_{y}\\ 0 & 0 & 1 \end{array}\right]$ is a camera matrix of intrinsic parameters, R is a 3 × 3 rotation matrix, and X,Y,Z are the ground coordinates in mm.

Both IR and RGB cameras suffer from distortion, mostly radial distortion and slight tangential distortion. This effect can be estimated based on Equation (2) [23]. The model illustrated three parameters for radial distortion ($k_{1},k_{2},k_{3}$) and two parameters for tangential distortion $\left(p_{1},p_{2}\right)$. As the RGB camera, the one used in this structure sensor is the smartphone’s camera, is expected to produce high distortion in both radial and tangential directions, we illustrate the full model parameters in our model [29].

$$u^{\prime }=u\left(1+k_{1}r^{2}+k_{2}r^{4}+k_{3}r^{6}\right)+\left[2p_{1}v+p_{2}\left(r^{2}+2u^{2}\right)\right]$$

(2) $$v^{\prime }=v\left(1+k_{1}r^{2}+k_{2}r^{4}+k_{3}r^{6}\right)+\left[2p_{2}u+p_{1}\left(r^{2}+2v^{2}\right)\right]$$

where $r^{2}=u^{2}+v^{2}$. By retyping Equation (1) as image point vector p and ground point vector *P*, where p=[u v 1] and P=[X Y Z 1].

(3) s·p=K[R|t][P]

This equation can be applied to RGB and IR cameras. The two sensors collected the same scene for an ordinary checkerboard. Therefore, by knowing the ground coordinates and image coordinates of the checkerboard corners, one can estimates the internal K and external R,t parameters for each camera using sufficient scenes.

For initial parameters estimation for K,R, and t, we use homography transform estimation which transforms the intrinsic and extrinsic matrix to one matrix called homography which can illustrate the relationship between the image point and the corresponding ground point in 3 × 3 matrix. If Z=0, then Equation (4) is simplified to:

(4) s·p=H·P

For eliminating the scale factor s, we can make a cross product for both sides by p. The resulting equation will be:

$$p\times s\cdot p=p\times \left(H\cdot P\right)=\left[\begin{array}{ccc} 0 & 0 & 0 \end{array}\right]$$

(5) $$H=\left[\begin{array}{ccc} h_{1} & h_{2} & h_{3} \end{array}\right]$$

Based on Equation (6), we can estimate the homography matrix known the image point and the corresponding ground point, by using Singular Value Decomposition (SVD) we can compute the homography matrix. Finally, with estimated homography matrix we can extract the internal and external parameters for each camera based on the characteristic of first and second column of R, which are orthonormal. Therefore, we can rewrite this formulae $h_{1}^{t}\cdot K^{-t}\cdot k^{-1}\cdot h_{2}=0$ as $h_{1}^{t}\cdot B\cdot h_{2}=0$, where $B=K^{-t}\cdot K^{-1}$. B is 3 × 3 matrix which contains only the internal parameters for camera. Based on characteristic of this matrix we can reduce the nine parameters to only six parameters. Thus, we can rewrite the last formulae as $h_{1}^{t}\cdot B\cdot h_{2}=L_{12}^{t}\cdot b=0$, where b is a vector which contains only six parameters, and

(6) $$L_{12}=\left[\begin{array}{c} h_{o1}\cdot h_{s1}\\ h_{o1}\cdot h_{s2}+h_{o2}\cdot h_{s1}\\ \begin{array}{c} h_{02}\cdot h_{s2}\\ h_{o3}\cdot h_{s1}+h_{o1}\cdot h_{s3}\\ \begin{array}{c} h_{o3}\cdot h_{s2}+h_{o2}\cdot h_{s3}\\ h_{o3}\cdot h_{s3} \end{array} \end{array} \end{array}\right]$$

Subscription of o and s refer to first and second column of H matrix, respectively. From this equation, we can compute the vector b, which summarizes the internal parameters for the camera using SVD. After that, we can reconstruct the intrinsic matrix K for the camera and then compute the rotation matrix and translation vector from homography matrix and intrinsic matrix. The output values for rotations and translations as well as the internal parameters can be used as initial value for refinement stage. The cost function to be minimized is:

(7) $$\min\left\{{∥P_{mn}-\left\{P_{mn}\cdot \left[\begin{array}{c} R_{n}\\ T_{n} \end{array}\right]\cdot \left[K\right]\right\}∥}_{color}^{2}+{∥P_{mn}-\left\{P_{mn}\cdot \left[\begin{array}{c} R_{n}\\ T_{n} \end{array}\right]\cdot \left[K\right]\right\}∥}_{IR}^{2}\right\}$$

where m is the point number and n is the scene number.

**Figure 3:**
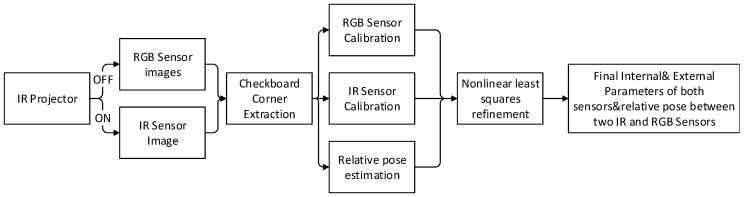
Methodology for RGB-D cameras Calibration.

As shown in Figure 3, the difference between the RGB camera and the depth camera lies in their methods of data collection. Due to the specific mechanics of the hardware, the sensor cannot collect the IR images and RGB images at the same time. The RGB camera collects RGB images all the time, but the data collected by the depth sensor depends on the status of the IR projector. When the IR projector is switched on, the IR camera collects the depth data for the scene. When the IR projector is switched off, the IR camera captures an ordinary image, which is similar to the RGB image, but on the IR band. The depth images on the IR band are used for the calibration process.

The result for this method is the full set of calibration data for external and internal parameters as well as the relative pose between RGB camera and IR camera. These parameters used in the robust geometric registration process are shown in Section 3.4.

### 3.3. Refined Relative Motion Estimation for RGB Images Sequence

The task of relative pose estimation, which is done by computing consistent feature matches across multiple images, presents a classic problem. Numerous algorithms have been proposed to solve this issue [27,30,31,32]. Normally, two steps would be involved in the relative motion estimation: key-point detection and matching, camera pose estimation. In our work, we add a refined outlier rejection method to eliminate the false matches by using the depth information as a reference and the pose derived from the ICP algorithm as a priori information. In the following subsections, we summarize the steps in the motion estimation algorithm.

#### 3.3.1. False Matches Rejection Method

The SiftGPU detector (which is an implementation of SIFT [33] for GPU) is used for image feature detection. SiftGPU processes pixels in parallel to build Gaussian pyramids and to detect DoG key points. Based on the GPU list generation [34], SiftGPU then uses a GPU/CPU mixed method to efficiently build compact key point lists. Finally, the key points are processed in parallel to obtain their orientations and descriptors. Typically, thousands of SIFT key points can be detected in each RGB image from RGB-D sensors with 640 × 480 pixels. Based on the local descriptor of each key point, we can use the approximate nearest neighbors package for feature matching [35].

However, several false matches still exist after the feature detection and feature matching processes. We therefore introduce an effective method to reduce the outliers by combining the depth information and the initial camera poses between frames from the RGB-D sensor plus RANSAC (RANdom Sample Consensus).

Supposing feature matches dataset is $D=\left\{p_{j}^{i}|1\leq i\leq N,\;1\leq j\leq M\right\}$, each point $p^{i}\left(1\leq i\leq N\right)$ can be detected in *M* frame, which are respectively represented with $\left\{p_{1}^{i},p_{2}^{i},\ldots p_{M}^{i}\right\}$. As mentioned by Khoshelham et al. (2013) [19], a correction of the depth image pixels should be used to align the depth image with the color image. In this paper, the discrepancy is calibrated by using markers that can be measured in the depth image as well as in the color image. The corresponding points from the infrared frame and the RGB image can be obtained and the affine transformation for the depth image pixels is calculated with a least-squares process. Therefore, corresponding to the points set on 2D images, 3D coordinates for each feature points can be obtained from depth image according to Equation (1), which can be represented as $C=\left\{q_{j}^{i}|1\leq i\leq N,\;1\leq j\leq M\right\}$. The basic idea of the method is to acquire feature matches from the RGB images, to compute global 3D coordinates of every feature points based on the initial pose parameters generated by RGB-D sensor. For each feature matches in D, we adopt RANSAC method to iterate and obtain the optimal 3D coordinates of feature matches $C_{1}=\left\{q^{\prime i}|1\leq i\leq N\right\}$. Then, $q^{i}\left(1\leq i\leq N\right)$ can be backprojected to the target frames and the corresponding image points set $D_{1}=\left\{p1_{j}^{i}|1\leq i\leq N,\;1\leq j\leq M\right\}$ can be obtained. Finally, the residual error between the original image point and the corresponding backprojected point in the image space $E=\left\{d_{j}^{i}|1\leq i\leq N,\;1\leq j\leq M\right\}$ is compared with the distance threshold $R.E._{uv}$ (the value of $R.E._{uv}$ depends on the accuracy of the initial pose from RGB-D sensors) and a point is recognized as an outlier whenever the residual error is greater than $R.E._{uv}$. In Algorithm 1, for each match, if the remaining image points, n2DInlier, is bigger than 3, it is recognized as inlier, otherwise it is outlier.

**Algorithm 1** False matches rejection combining depth information + RANSAC**Input:**
$D=\left\{p_{j}^{i}|1\leq i\leq N,\;1\leq j\leq M\right\}$: feature matches set in image space; $C=\;\left\{q_{j}^{i}|1\leq i\leq N,\;1\leq j\leq M\right\}$: 3D points corresponding to feature matches; $d_{XYZ}$: distance threshold in object space for RANSAC iterations; $d_{uv}$: distance threshold in image space**Output:** number of inliers: n2DInlier, Inliers: F = $\left\{p\prime_{j}^{i}|1\leq i\leq N,\;1\leq j\leq M\right\}$
1. F=∅2. **For**
*i* = 1→n **do**3.  Iterations = 0, $n3DInlier_{max}=0$, G = ∅
4.  **While** Iterations <= MaxIterations **do**5.   n3DInlier = 06.   Randomly select 5 feature points in current feature matches from $q^{i}$, compute the mean value of 3D coordinates $q\prime^{i}$
7.   **For**
$q^{i}\in \left\{q^{i}\right\}$
**do**8.    **If**
$∥q^{i}-q^{\prime i}∥$ < $R.E._{XYZ}$
**then**9.      n3DInlier = n3DInlier + 110.     $3\mathrm{DInliers}=\;3\mathrm{DInliers}\cup^{\text{​}}\left\{q^{i}\right\}$11.     **End if**12.   **End for**13.   **If** n3DInlier > $n3DInlier_{max}$
**then**14.    $n3DInlier_{max}=\;$n3DInlier15.    G=3DInliers16.   **End if**17.  **End while**18.  $q^{\prime i}=\frac{\sum_{n=0}^{n3DInlier_{max}}G^{n}}{n3DInlier_{max}}$19.  n2DInlier = 0, f=∅20.  **For** j = 1→m **do**21.   Let $q^{\prime i}$ backproject to j-th Frame, obtain the backprojected image point $p1_{j}^{i}$22.   **If**
$∥p_{j}^{i}-p1_{j}^{i}∥$ < $R.E._{uv}$
**then**23.    $f=f\cup^{\text{​}}p_{j}^{i}$24.    n2DInlier=n2DInlier+125.   **End if**26.  **End for**27.  **If**
n2DInlier≥3
**then**28.   $F=F\cup^{\text{​}}f$29.  **End if**30. **End for**31. **Return**
n2DInlier,F

It should be noted that due to the limitation in measurement distance of the RGB-D sensor, it is impossible to find all of the corresponding points from the depth image. Therefore, the outlier rejection method can only be used within a certain range (within 8 m) and the thresholds $R.E._{uv}$ and $R.E._{XYZ}$ differ with the increasing of measurement distance.

#### 3.3.2. Camera Pose Estimation for RGB Images Sequence

As frame rate to RGB-D sensors speed ratios are often higher than necessary, not all of the RGB images need to be processed, so choosing the right frames requires careful consideration. Camera baselines and overlap between images are highly important for robust 3D reconstruction. Short baselines usually induce larger measurement errors than those produced by the long baselines [36]. Therefore, the selection criteria must guarantee both enough baseline and sufficient overlap between the candidates and the previous key frame.

In this paper, the initial pose from RGB-D sensor is employed to ensure enough baseline by computing Euclidean distance between two frames. Besides, we use the correspondence ratio $R_{C}$ (the ratio of the number of frame-to-frame point features to the total number of point features considered for correspondence) defined by [37] to ensure sufficient overlap between the candidates and the previous key frame. The image is selected as a key frame whose ratio of feature point to correspondence is less than 90% and the baseline $B_{l}$ between the candidates and the previous key frame is greater than 10 cm. If the ratio is greater than 90% or the baseline is less than 10 cm, we consider the next frame as candidates until find the next key frame.

(8) $$\{\begin{array}{c} R_{c}<90\%\;\&\;B_{l}>10cm\;Key\;frame\\ R_{c}>90\%\;or\;B_{l}<10cm\;Ignored\; \end{array}$$

Finally, we then robustly estimate a fundamental matrix between key frames $F_{n-1}$ and $F_{n}$ by using the five-point algorithm proposed by Nistér [2] and the RANSAC method [38]. Then, the rotation $R_{c}$ and translation $t_{c}$ are recovered by matrix factorization. This minimization problem is solved with the Levenberg–Marquardt nonlinear optimization [39], and $R_{c}$ and $t_{c}$ are further refined. The corresponding 3D coordinates of feature matches can be calculated with space intersection.

### 3.4. Robust Geometric Registration of RGB and Depth Models

Since the geometry of RGB images (rotation *R* and translation *T* of each RGB image, 3D coordinates of feature matches) obtained in Section 3.3 can only be recovered up to a scale factor and the coordinates system is different from that of depth sensor, the robust geometric registration method aims to integrating geometry of RGB images and depth geometry according to a global scale recovery and rigid transformation recovery method. Tie points are obtained on the RGB images based on the image matching algorithm in Section 3.3.1. The 3D coordinates of feature matches can be derived from the space intersection using the recovered RGB image pose. There would be discrepancies between the RGB pose-derived object coordinates and the ground truth obtained from depth image according to the camera model for depth images. First, a global optimization model is employed to improve the accuracy of the camera pose, decreasing the inconsistencies between the depth frames. Then, a global scale for RGB geometry is recovered by computing the distance ratio between the point pairs of RGB pose-derived points and depth-derived points and the rigid transformation between the two sets of corresponding 3D points is calculated to ensure that they are aligned. Ultimately, the inconsistencies between two sets of corresponding 3D points is eliminated with the recovered scale and rigid transformation.

#### 3.4.1. Camera Model for Depth Images

By knowing the internal parameters and distortion of depth camera by camera calibration, we can compute the object coordinates $X_{c},\;Y_{c},Z_{c}$ in the camera coordinate system from the image space as follows:

$$X_{c}=\frac{D}{f_{x_{D}}}\;\left(u^{\prime }-c_{x_{D}}\right)$$

(9) $$Y_{c}=\frac{D}{f_{y_{D}}}\;\left(v^{\prime }-c_{y_{D}}\right)$$

$$Z_{c}=D$$

where $f_{xD},\;f_{yD}$ are the focal length of the depth camera, $c_{xD},\;c_{yD}$ are the image center of the depth image, and $u^{\prime },\;v^{\prime }$ are the image coordinate corrected by distortion parameters.

**Figure 4:**
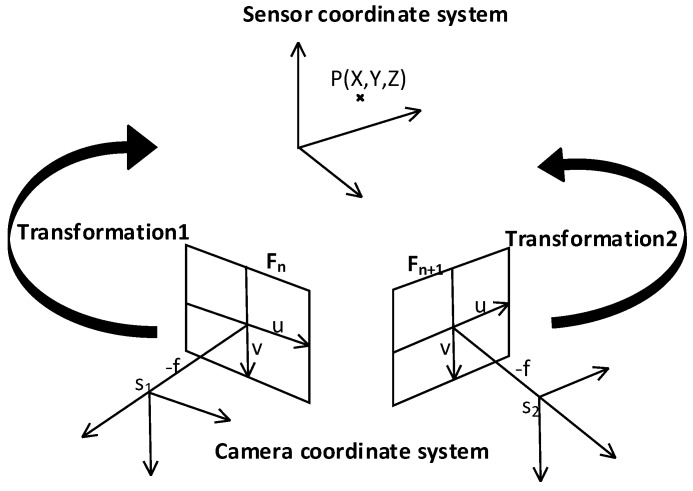
Relationship between the camera and the sensor coordinate systems.

A rigid body transformation relates points $\tilde{X}\sim {\left[\begin{array}{ccc} X & Y & \begin{array}{cc} Z & 1 \end{array} \end{array}\right]}^{T}$ in the sensor coordinate system of the referenced frame to points $\tilde{X_{C}}\sim {\left[\begin{array}{ccc} X_{C} & Y_{C} & \begin{array}{cc} Z_{C} & 1 \end{array} \end{array}\right]}^{T}$ in the camera coordinates of the current frame. This transformation can be written as

(10) $$\left[\begin{array}{c} X\\ Y\\ \begin{array}{c} Z\\ 1 \end{array} \end{array}\right]=\left[\begin{array}{c} \begin{array}{cc} R_{D} & t_{D} \end{array}\\ \begin{array}{cc} 0 & 1 \end{array} \end{array}\right]\left[\begin{array}{c} X_{C}\\ Y_{C}\\ \begin{array}{c} Z_{C}\\ 1 \end{array} \end{array}\right]$$

where $R_{D}$ is the rotation matrix from current frame to the referenced frame, $t_{D}$ is the translation matrix from current frame to the referenced frame, and X, Y, Z are the real object coordinates in the 3D scene. Figure 4 shows the relationship between the camera and the sensor coordinate systems.

#### 3.4.2. Joint Optimization Model for Poses of Depth Camera

The RGB-D camera uses the ICP algorithm for depth alignment. An initial relative camera pose for each frame can thereby be obtained. However, errors in alignment between depth frames and noise in depth information cause the camera pose to drift over time, especially when the camera follows a long trajectory. Therefore, a global optimization model is used for decreasing the inconsistencies between frames in advance. All of the feature matches in Section 3.3.1 and the initial camera pose obtained from the ICP alignment are involved in the model. Supposing the total number of the frame pairs is M and for each frame pair a, b, the total number of the point pairs is N. The corresponding features matches dataset {PP} can be represented as:

(11) $$PP=\left\{p_{j}^{a},\;p_{j}^{b}|1\leq j\leq N\right\}$$

Therefore, the discrepancy between two point pair can be represented as follows:

(12) $$D_{j}^{ab}=∥(R_{a}p_{j}^{a}+t_{a})-\left(R_{b}p_{j}^{b}+t_{b}\right)∥$$

where $\left\{R_{a},t_{a}\right\}$ and $\left\{R_{b},t_{b}\right\}$ are the initial rotation and translation matrix of the frame a, b, respectively. For the whole scene, the cost function can be written as Equation (11) and a least square solution is used to minimize the error iteratively. The global optimization model ultimately improves the accuracy of the camera pose, decreasing the inconsistencies between the depth frames.

(13) $$\min\left\{\overset{M}{\underset{i=0}{\sum }}\overset{N}{\underset{j=0}{\sum }}D_{j}^{ab}\right\}$$

#### 3.4.3. Global Scale Recovery for RGB Images

**Figure 5:**
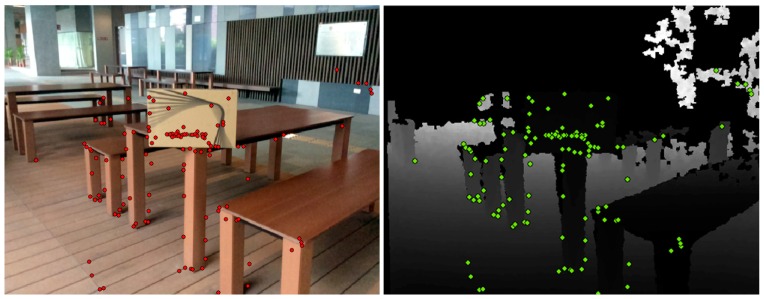
(**left**) Feature matches from an RGB image; and (**right**) feature matches on the corresponding depth image.

Based on recovered RGB images poses, the 3D coordinates for each tie point can be obtained by a space intersection. As a control, we select the registered depth frame that possesses the greatest number of corresponding points between RGB image and depth image. As shown in Figure 5, for each feature match located in the RGB image, the image coordinates can be obtained and the corresponding depth value can be extracted from the registered depth image. The points that have no depth value are discarded. The ground truth of each point can be calculated from Equations (9) and (10).

Two sets of 3D points, $P_{C}=\left\{P^{i}|1\leq i\leq N\right\},\;P_{D}=\left\{P^{\prime i}|1\leq i\leq N\right\}$ can be obtained from RGB images and depth images, respectively. The *P_C_* set is obtained from the space intersection of the RGB images, and the *P_D_* set is obtained from the depth images. Then, the relative scale S can be determined from the distance ratio between the point pairs of the two points sets $P_{C},\;P_{D}$, as follows:

(14) $$S=\frac{\sqrt{{\left(X_{P_{D}^{i}}-X_{P_{D}^{j}}\right)}^{2}+{\left(Y_{P_{D}^{i}}-Y_{P_{D}^{j}}\right)}^{2}+{\left(Z_{P_{D}^{i}}-Z_{P_{D}^{j}}\right)}^{2}}}{\sqrt{{\left(X_{P_{C}^{i}}-X_{P_{C}^{j}}\right)}^{2}+{\left(Y_{P_{C}^{i}}-Y_{P_{C}^{j}}\right)}^{2}+{\left(Z_{P_{C}^{i}}-Z_{P_{C}^{j}}\right)}^{2}}}\left(i!=j\right)$$

For a robustness test, a large number of scale ratios for point pairs are calculated at random, the Pau Ta Norm are used for outlier rejection, as in Equation (15). RANSAC is used to iterate and calculate the optimal scale value.

(15) $$\{\begin{array}{c} \left|S_{c}-\bar{S}\right|>3\sigma \;\left(outlier\right)\\ \left|S_{c}-\bar{S}\right|<3\sigma \;\left(inlier\right) \end{array}$$

where $S_{c}$ is mean value of 5 scale values selected at random, $\bar{S}$ is the median value of the scale set, and σ is the root-mean-square error of the scale set.

The global scale recovery method is presented in Algorithm 2. First, a set of scale values $F_{s}$ is calculated iteratively, and, in each iteration, the point pairs from $P_{C},\;P_{D}$ is selected at random. To find the optimal scale value, we iteratively apply Pau Ta Norm to the subset with 5 scale values selected from $F_{s}$ randomly, the scale subset with the biggest number of inliers $F\prime_{s}$ is returned and the proper scale is determined by the mean value of the inliers. The point sets from the space intersection of the RGB images are scaled to a new point set $P_{S}$, as follows:

(16) $$\left[\begin{array}{c} X_{P_{S}}\\ Y_{P_{S}}\\ \begin{array}{c} Z_{P_{S}}\\ 1 \end{array} \end{array}\right]=\left[S\right]\left[\begin{array}{c} X_{P_{C}}\\ Y_{P_{C}}\\ \begin{array}{c} Z_{P_{C}}\\ 1 \end{array} \end{array}\right]$$

**Algorithm 2** Global Scale Recovery for RGB images + Pau Ta Norm and RANSAC**Input:**
$P_{C}=\left\{P^{i}|1\leq i\leq N\right\}$: 3D points from RGB images; $P_{D}=\left\{P^{\prime i}|1\leq i\leq N\right\}$: 3D points from depth images**Output:** S1. $F_{s}=\emptyset$2. **For**
*i* = 1→$\frac{\left[N\times \left(N-1\right)\right]}{2}$
**do**3.  Randomly select 2 points from $P_{C},P_{D}$, compute scale value S4.  $F_{s}=F_{s}\cup^{\text{​}}S$5. **End for**6. Compute the mean value $\bar{S}$ and the root-mean-square error σ
7. $nInlier_{max}=0,$ S = 08. **While** Iterations <= MaxIterations **do**9.   nInlier=0, $F\prime_{s}=\emptyset$10.  Randomly select 5 scale values from $F_{s}$, compute the mean value $S_{c}$11.  **For** i = 1→$\frac{\left[N\times \left(N-1\right)\right]}{2}$
**do**12.   **If**
$\left|S_{c}-\bar{S}\right|<3\sigma$
**then**13.    nInlier = nInlier + 114.    $F\prime_{s}=F\prime_{s}\cup^{\text{​}}S_{c}$15.   **End if**16.  **End for**17.  **If** nInlier > $n3DInlier_{max}$
**then**18.   $nInlier_{max}=\;$nInlier19.   $S=\frac{\sum_{n=0}^{{\mathrm{nInlier}}_{max}}F\prime_{s}^{n}}{nInlier_{max}}$20.  **End if**21. **End while**22. **Return**
S

#### 3.4.4. Rigid Transformation Recovery

After scale recovery, it is necessary to find the optimal rotation and translation between the two sets of corresponding 3D points to ensure that they are aligned. We compute the rigid transformation matrix with Besl’s method [40]. This solution can be used for a dataset of any size, as long as there are at least three corresponding points. A least square solution is used to minimize the error as in Equation (17).

(17) $$\min\left(\overset{N}{\underset{i=1}{\sum }}{∥RP_{s}^{i}+t-P_{D}^{i}∥}^{2}\right)$$

The method based on a Besl’s rigid transformation estimator plus RANSAC is presented in Algorithm 3. In each iteration, we randomly select 5 pairs of corresponding points from $\left\{P_{s}\right\}$ and $\left\{P_{D}\right\}$, the current rigid transformation *R*’, *t*’ can be calculated with Besl’ method. The threshold value used for outlier rejection is determined by the initial pose accuracy obtained from depth sensor. RANSAC method is used to iterate and seek the optimal corresponding points set. An iterator is used to loop through the point pairs in $\left\{P_{s}\right\}$ and $\left\{P_{D}\right\}$, it is recognized as inlier when the distance between $P\prime_{i}$ and $R^{\prime }P_{s}^{i}+t\prime$ is less than Threshold. The corresponding points set with the most inliers is used to compute the final rigid transformation matrix R, t.

**Algorithm 3** Rigid Transformation Recovery**Input:**
$P_{s}=\left\{P^{i}|1\leq i\leq N\right\}$: scaled 3D points from RGB images; $P_{D}=\left\{P^{\prime i}|1\leq i\leq N\right\}$: 3D points from depth images**Output:** best transformation estimation (R, t)1. $nInlier_{max}=0$, Iterations = 0, F=∅2. **While** Iterations <= MaxIterations **do**3.  nInliers=0, Inliers=∅4.  Randomly select 5 pairs of corresponding points from $\left\{P_{s}\right\}$ and $\left\{P_{D}\right\}$, use Besl’s method to compute the rigid transformation *R*’, *t*’5.  **For**
$P_{i},\;P\prime_{i}\in \left\{P_{s}\right\},\;\left\{P_{D}\right\}$
**do**6.   **If**
$∥P\prime_{i}-(R^{\prime }P_{s}^{i}+t^{\prime })∥<Threshold$
**then**7.    nInlier = nInlier + 18.    Inliers=Inliers∪{i}9.   **End if**10.  **End for**11.  **If** nInlier > $nInlier_{max}$
**then**12.   $nInlier_{max}=\;$nInlier13.   F=Inliers14  **End if**15.  Iterations = Iterations + 116. **End while**17. $\left(R,t\right)=\underset{R,t}{\min}\sum_{i\in F}{∥RP_{s}^{i}+t-P_{D}^{i}∥}^{2}$18 **Return**
(R, t)

By knowing the scale factor *S* and the rigid transformation R, t between the 3D coordinates of RGB scene and that from depth scene, the model generated from RGB images can be registered to the coordinates system of depth model with Equation (18).

(18) $$\left[\begin{array}{c} X\\ Y\\ \begin{array}{c} Z\\ 1 \end{array} \end{array}\right]=\left[\begin{array}{cc} R & t\\ 0 & 1 \end{array}\right]\left[S\right]\left[\begin{array}{c} X_{P_{c}}\\ Y_{P_{c}}\\ \begin{array}{c} Z_{P_{c}}\\ 1 \end{array} \end{array}\right]$$

Finally, the absolute camera trajectory of RGB images sequence $R_{a}$, $T_{a}$ can be written as Equation (19), which can be used for dense matching with the CMPMVS tool. CMPMVS tool is a multi-view reconstruction software. The input to this software is a set of perspective images and camera parameters (internal and external camera calibrations). The output is a textured mesh of the rigid scene visible in the images [41]. Then, the dense model generated from RGB images sequence can be matched with the 3D model obtained from the depth images.

(19) $$\left[\begin{array}{cc} R_{a} & t_{a}\\ 0 & 1 \end{array}\right]=\left[\begin{array}{cc} R_{c} & t_{c}\\ 0 & 1 \end{array}\right]{\left(\left[\begin{array}{cc} R_{D} & t_{D}\\ 0 & 1 \end{array}\right]\left[\begin{array}{cc} R & t\\ 0 & 1 \end{array}\right]\left[S\right]\right)}^{-1}$$

## 4. Experiments and Results

### 4.1. Benefit of Joint Optimization Model

We first evaluated our joint optimization method with the publicly available RGB-D benchmark provided by [42]. The public RGB-D benchmark dataset is used to assess the accuracy of the camera trajectory and the results is compared with the state-of-the-art methods. They contain ground truth information for camera poses in terms of time-series. Absolute trajectory error is used for trajectory estimation and comparative estimation.

**Figure 6:**
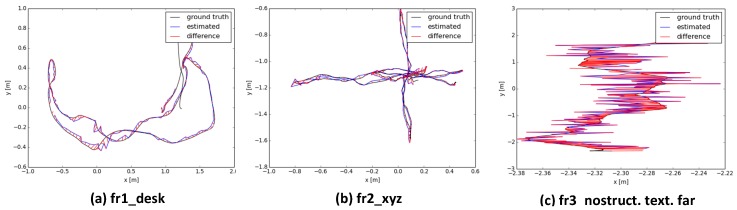
Estimated trajectories compared against ground truth trajectories.

Three sets of publicly available datasets are used for accuracy evaluation. Figure 6 shows the estimated camera trajectories compared against the ground truth trajectories. As shown in Table 1, for the datasets with structure, like fr1_desk and fr2_xyz, our method can achieve median and maximum absolute trajectory accuracy within 3 cm and 10 cm, respectively. Difficult scenes contain only little geometric structure but with fine texture like fr3_nostruct.tex.far sequences, the proposed joint optimization method can also yield only moderate trajectory drift, about 3.2 cm in median and 7 cm in maximum.

Table 1 also shows the comparison of median (maximum) absolute trajectory error for joint optimization between our method and several state-of-art registration methods including 3D-NDT method [43], Warp from OpenCV [44] and Fovis method [45]. The best results are marked in bold. Except for the maximum error in fr2_xyz sequences, our approach outperforms the other methods both in the median error and the maximum error. In the second case, all methods yield similar accuracy because of the rich texture information, and our method achieves the best median result because of the robust false matches rejection method in Section 3.3.1. Especially for the scene with no geometric information, our method performs much better than three others.

### 4.2. Experiments of Robust Geometric Registration

#### 4.2.1. Datasets

In this section, we carry out the field tests to validate the feasibility and effectiveness of the proposed enhanced RGB-D mapping method. Two sets of data were collected, using the structure sensor attached to an iPad Air. We conducted a precise camera calibration for this device and the camera calibration results including the internal parameters and distortion parameters are shown in Table 2.

**Figure 7:**
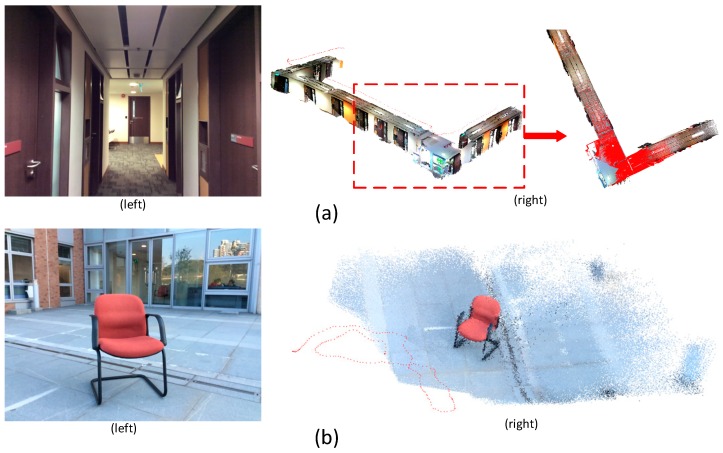
(**a**) Dataset captured along a corridor; and (**b**) dataset captured in the outside environment.

The first dataset is used to deal with the sequence captured along a corridor. The two images in Figure 7a (left) shows a sample RGB frame. The 3D model generated from depth images based on the ICP + Global optimization sequential alignment, the corresponding camera trajectory marked with red points and a top view of the 3D model overlaid on a laser scan point cloud are shown in Figure 7a (right). The whole length of the camera trajectory was about 26.5 m, and it contained 305 registered frames. To further investigate the performance of the proposed methodology in an outside environment, as shown in the RGB image in Figure 7b (left), one chair was placed in front of the wall and the dataset was collected by walking around the chair. A total of 196 registered frames were obtained. The corresponding 3D scene generated from the depth images shown in Figure 7b (right).

#### 4.2.2. Experimental Results and Analysis

To further thoroughly evaluate the benefits of global optimization model, the accuracy of the camera poses is determined by computing the discrepancies in the contiguous frames. Instead of placing targets on the ground, the exact truth poses are obtained through frame alignment manually. To reduce the time complexity, only the truth rotation and translation between the adjacent key frames are obtained as referenced, the translational error and the angular error of the sequential alignment can be obtained by comparing with the ground-truth poses. As can be seen in Table 3, by combining ICP and global optimization, it achieves accuracy which is superior to using the ICP algorithm only. In the ICP algorithm, the alignment accuracy highly depended on the geometric information in the adjacent frames. However, in the corridor experiment, it provides little geometric information, and the frames mainly contain several single flat walls. It is reasonable that global optimization model can improve the alignment accuracy due to involving additional RGB information.

In addition, the corridor model generated from the structure sensor is compared with a laser scan point cloud. As shown in Figure 6a (right), these two models can match well in both horizontal and vertical direction. To evaluate the absolute accuracy of the coordinator model, some key point pairs are selected from the sensor model and the laser scanner and the distance between two point pairs selected at random is calculated. The average distance errors are shown in Table 3. Similar with two others, ICP + Global Optimization can achieve the absolute accuracy to centimeter level, which is higher than that of the ICP algorithm.

After applying global optimization for the pose of depth camera, we implement the robust geometric registration m to register the 3D model based on image-based modeling method to the model generated from depth images, and then the results is compared with the model totally generated from depth images. Check points are selected from the results of feature matching. For each check point, two sets of object coordinates can be obtained from the image-based model and the model from depth respectively. Then, we achieved a relative accuracy assessment of the obtained result through the root mean square error (RMSE) of the discrepancies of each check points in the object space. It should be noted that only the depth within 3 m of the depth frame is used for accuracy assessment.

**Figure 8:**
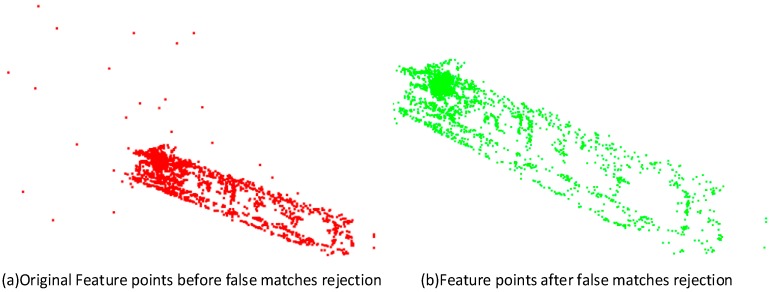
False matches rejection for corridor model.

In the corridor experiment, 172 frames are selected as key frames and then are used for 3D modeling. The feature matches in the key frames are first checked with the false matches rejection method, the corresponding $R.E._{uv}$ and $R.E._{XYZ}$ are set at 10 pixels and 0.2 m, respectively, according to the initial accuracy of the camera pose. Figure 8 shows the comparison of feature matches in the corridor images. The original 3980 feature matches are obtained after using a traditional RANSAC false matches rejection method. In RANSAC, the threshold for estimating *F* matrix is 2, and the threshold for estimating *H* matrix is 4. The maximum iterations in RANSAC is 1000. In this experiment, 42 more false matches can be rejected by using the refined false matches rejection method in this paper. Then, 432 feature matches identified from the first frame are used for geometric registration. Due to the measurement distance limitation of depth sensor, 1302 feature points with depth value within 3 m are used to check the performance of geometric registration.

The performance of geometric registration approach is evaluated in object space. The 1302 check points are compared based on the object coordinates from depth information and the transformed coordinates from RGB sequences. Table 4 lists registration results including the recovered scale, rigid transformation and the statistics of discrepancies between two models after geometric integration. As Table 4 shows, the discrepancies between the scene from depth images and the scene from RGB images can accurate to centimeter-level (within 3 cm) in all the three directions. This indicates that the geometric inconsistencies between the geometry of RGB images and depth images are nearly eliminated.

**Figure 9:**
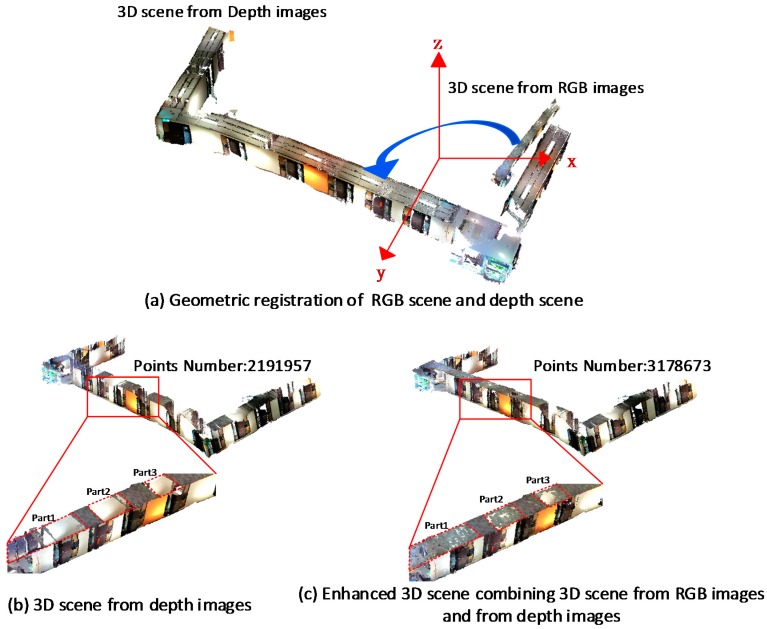
Results of geometric registration for corridor model.

In Figure 9a, the 3D scene from RGB images is first transformed to the coordinate system of depth scene based on the recovered scale and rigid transformation parameters. Figure 9b shows the original 3D scene totally generated from depth image. Although all of the depth frames were used for scene modeling, significant details are lost, especially on the ceiling and the floor. Figure 9c shows the enhanced 3D scene combining 3D scene from RGB images and from depth images after geometric registration. The vertices have significantly increased from about two million to three million. In Figure 9b, the broken regions are marked with red dotted borders. As expected, the scene detail in the corresponding regions is enriched significantly after geometric registration shown in Figure 9c. It means that the model generated from the corresponding RGB images can be a good supplement to the model from depth images.

For the chair model collected outside, 86 frames are selected as key frames. The corresponding $R.E._{uv}$ and $R.E._{XYZ}$ parameters for false matches rejection are set at 3 pixels and 0.05 m, respectively, due to high accuracy of the camera pose. The 6293 feature matches were obtained and 38 more false matches are rejected. The 246 feature points are used for geometric registration. The performance of the geometric registration is examined with 1278 check points.

The performance of geometric registration approach was evaluated in object space. The 1278 check points were compared based on the object coordinates from depth information and the transformed coordinates from RGB images. Table 4 lists registration results including the recovered scale, rigid transformation and the statistics of discrepancies between two models after geometric integration. As Table 4 shows, the geometric registration accuracy can obtain an accuracy of less than 2 cm in all three directions. Since the model from depth images is used as reference for geometric registration accuracy evaluation and the check points are selected from different frame, the consistency between depth frames can directly influence the performance of the registration method. The inconsistency between frames grows with the distance of the trajectory due to error propagation during frames alignment. In the corridor experiment, the length of the camera trajectory is much higher than that of the outdoor experiment, the global consistency of the scene is worse than that of the scene of the outdoor. The better consistency results in higher accuracy of the initial pose parameters. Therefore, the geometric registration accuracy should be higher in the chair scene than that in the corridor scene.

**Figure 10:**
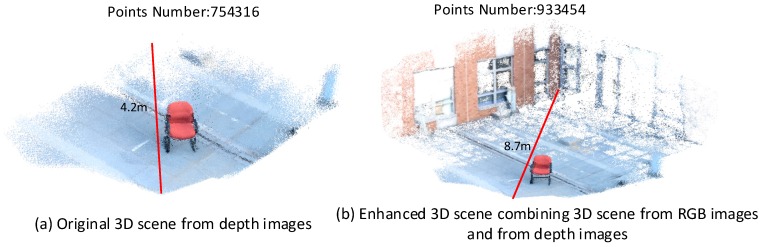
Results of geometric registration for chair model.

Figure 10a,b shows the original 3D scene generated from depth image and the enhanced 3D scene combining 3D information from RGB images and from depth images after geometric registration, respectively. Only a close-range scene with about 4.2 m maximum length can be obtained from the depth images. As the far-range model generated from the RGB images is added to the original 3D scene from depth image, the vertices number have a significant increase from 754,316 to 933,454 and the measurement distance can be extended to about 9 m. In this case, the information from the RGB image sequences both enriched the details for the close-range model from the depth images and greatly broadened the modeling range of the RGB-D camera.

## 5. Summary and Conclusions

The key issues that we encountered when using RGB-D sensors to produce 3D models are the limited measurement distance and the limited field of view. Other key insights of this investigation are that existing ICP frame matching techniques are not sufficient to provide robust visual odometry with these cameras; and a tight integration of depth and color information can yield robust frame matching and global optimization. We first presented a global optimization model for camera poses improvement that takes advantage of the richness of information contained in RGB images. Then we have presented a novel approach for the geometric integration of depth scene and RGB scene to enhance the mapping system of RGB-D sensors for detailed 3D modeling of large indoor environments. The 3D scene produced from the RGB images is innovatively used as supplement to the 3D scene produced by the depth sensors, which can not only enhance scene details where lack of depth information, but can also broaden the modeling range of RGB-D sensors. At the calibration stage, we employ a precise calibration method to obtain the full set of external and internal parameters as well as the relative pose between RGB camera and IR camera. In order to avoid false matches as much as possible, features extracted from RGB-D image are checked with a refined false matches rejection method. Based on the robust geometric registration method, the global scale of RGB camera motion and the rigid transformation between the RGB scene and depth scene is automatically recovered.

The benefit of the proposed global optimization method is firstly evaluated with the publicly available benchmark datasets collected with Kinect. Absolute trajectory error is used for trajectory estimation and comparative estimation. Then, we demonstrate the performance of the proposed robust geometric registration approach with results obtained when dealing with the dataset collected in inside and outside environments. The performance of the proposed enhanced mapping method is evaluated from two perspectives, the absolute accuracy of the sensor model and the relative registration accuracy between model from depth and RGB images.

Despite these encouraging results, our system has several shortcomings. The current implementation of the enhanced mapping system is not real-time. The global optimization model can handle up to about 200 frames, but we believe the model can be improved through proper algorithm optimization. The next step of this research is to concentrate on larger and more complicated environment and extend the system to implement a full modeling approach including real-time processing and mesh reconstruction.

## Figures and Tables

**Table 1:** Comparison of median (maximum) absolute trajectory error in mm for joint optimization on RGB-D sequences of the Freiburg Benchmark Dataset, best results in bold.

Datasets	Ours		3D-NDT		Warp		Fovis	
	Median	Max	Median	Max	Median	Max	Median	Max
**fr1_desk**	**2.2**	**9.7**	47.8	26.6	6.2	147	6.3	34.2
**fr2_xyz**	**1.2**	9.1	14	18	2	**8.8**	1.9	9.9
**fr3_nostruct. text. far**	**3.2**	7	18.6	74.6	19.2	246	20.8	101.5

**Table 2:** Calibration results of the IR camera and RGB camera.

IR Sensor	Focal length (pixels)	f_xD_	580 ± 3.49
		f_yD_	581 ± 3.27
	Principal point (pixels)	c_xD_	331.59 ± 1.57
		c_yD_	236.59 ± 1.98
	Distortion	K_1D_	−0.0075 ± 0.0188
		K_2D_	1.7812 ± 0.3383
		P_1D_	−0.0047 ± 0.0009
		P_2D_	0.0017 ± 0.0013
		K_3D_	−8.7810 ± 1.95
RGB Sensor	Focal length (pixels)	f_xC_	570.63 ± 3.43
		f_yC_	570.96 ± 3.20
	Principal point (pixels)	c_xC_	319.84 ± 1.55
		c_yC_	244.96 ± 2.01
	Distortion	K_1C_	−0.0378 ± 0.0209
		K_2C_	−0.5221 ± 0.3959
		P_1C_	−0.0025 ± 0.0007
		P_2C_	−0.0014 ± 0.0010
		K_3C_	3.9233 ± 2.3220

**Table 3:** Sequential alignment comparison with different method.

Method	Avg. Translational Error (m)		Avg. Angular Error (deg)		Avg. Distance Error (m)
	Corridor Experiment	Chair Experiment	Corridor Experiment	Chair Experiment	Corridor Experiment
ICP	0.236	0.143	3.563	1.724	0.265
ICP + Global Optimization	0.068	0.032	2.153	0.983	0.081

**Table 4:** Statistics on discrepancies in the object space between the model from depth and RGB images.

Dataset	Registration Results			RMSE of the Discrepancies from the Check Points		
	Scale Factor	Rigid Transformation		σ_x_ (m)	σ_y_ (m)	σ_z_ (m)
		*R*	*t*			
Corridor Model	2.796	174.997°	2.694	0.026	0.019	0.023
		4.657°	1.546			
		41.335°	−6.329			
Chair Model	1.075	174.915°	−0.955	0.015	0.014	0.012
		6.536°	−0.332			
		−21.312°	−3.304			
